# Long-term complete remission with belinostat in a patient with chemotherapy refractory peripheral t-cell lymphoma

**DOI:** 10.1186/1756-8722-6-69

**Published:** 2013-09-10

**Authors:** Peter Reimer, Shanta Chawla

**Affiliations:** 1 Klinik für Hämatologie, Internistische Onkologie und Stammzelltransplantation, Evangelisches Krankenhaus Essen-Werden gGmbH, Pattbergstr. 1-3, Essen 45239, Germany; 2 Spectrum Pharmaceuticals, 157 Technology Drive, Irvine, CA 92618, USA

**Keywords:** Peripheral T-cell lymphoma, Belinostat, HDAC inhibitor

## Abstract

Peripheral T/NK-cell lymphomas (PTCL) are rare malignancies with a poor prognosis. Due to the lack of randomised studies, standard therapy has not been established. First-line treatment with anthracycline-based polychemotherapy followed by consolidation with high-dose therapy and autologous stem cell transplant in responding patients has demonstrated good feasibility with low toxicity in prospective studies and is widely used in eligible patients. In relapsed and refractory patients, who are not candidates for transplant approaches, therapeutic options are limited and are usually palliative. Several new agents are currently under investigation to improve the outcome of PTCL in the first line and salvage settings. Belinostat, a histone deacetylase (HDAC) inhibitor, has demonstrated broad antineoplastic activity in preclinical studies, and promising results in advanced relapsed/refractory lymphomas.

Here, we report the case of a 73 year old patient with heavily pre-treated refractory PTCL in complete remission with belinostat for 39 months.

## Background

Peripheral T-cell lymphomas (PTCL) are rare malignancies representing approximately 10%-15% of all non-Hodgkin’s lymphomas (NHL) in Western countries [1-3]. With the exception of ALK (anaplastic lymphoma kinase) positive anaplastic large cell lymphoma, PTCL exhibits an aggressive course and patients have a poor outcome after conventional chemotherapy [4-7] with a median overall survival (OS) of 9 to 42 months [8-10]. Although anthracycline-based regimens are widely used as first-line therapy, their benefit has not been established prospectively in PTCL. In the absence of randomised trials, high-dose therapy with autologous stem cell transplantation is widely used as consolidation. For relapsed or refractory PTCL, autologous and allogeneic stem cell transplantation is the standard of care for eligible patients. However, chemotherapy-refractory patients are not good candidates for this approach. Several prospective trials have shown chemo-sensitivity to be the most important prognostic factor in the transplant setting and patients without at least a partial remission at transplantation have a poor outcome following either autologous or allogeneic stem cell transplantation. New approaches that include novel agents are urgently needed, and several different agents are currently under investigation to improve the outcome of patients with PTCL.

Belinostat is a potent inhibitor of histone deacetylase (HDAC) enzymes, which alter acetylation levels of histone and non-histone proteins. HDAC enzymes regulate multiple cellular processes and play a role in cancer cell proliferation and survival. Belinostat has demonstrated broad antineoplastic activity in preclinical studies and has been investigated in several Phase I and II studies in patients with solid and hematologic neoplasms. In 20 patients with refractory and relapsed PTCL, Pohlman et al. reported an overall response rate of 25% with a median duration of response of about 5 months [11]. Preliminary results of the large multicenter, open-label belinostat trial in relapsed and refractory PTCL were recently presented at the Annual Meeting of the American Society of Oncology [12].

We report the case of a 73 year old female patient with refractory PTCL, who remains in complete remission following treatment with belinostat for 39 months.

## Case presentation

**Figure 1:**
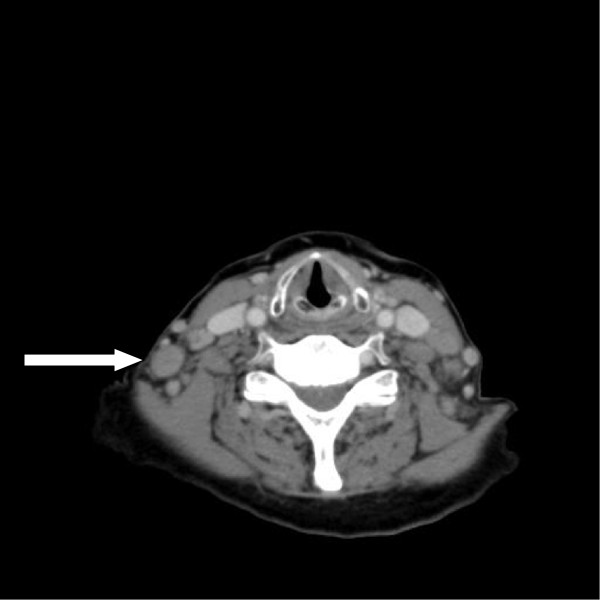
CT scan of the neck before starting belinostat therapy (January 2010) showing multiple lymph nodes mainly at the right site of the neck (arrow).

In June 2009, a 69 year old female patient was diagnosed with PTCL not otherwise specified (PTCL-NOS) after complaining of hoarseness. Staging procedures revealed a single manifestation with involvement of the epipharynx with no evidence of B symptoms (Ann Arbor stage IE A). The international prognostic index (IPI) and the prognostic index for T-cell lymphoma (PIT) were low (age above 60 years as the only risk factor). The patient was otherwise healthy with no relevant concomitant diseases. Following two cycles of chemotherapy with CHOP-21 (cyclophosphamide 750 mg/m^2^ day 1, vincristine 1.4 mg/m^2^ day 1, doxorubicin 50 mg/m^2^ day 1, and prednisolone 100 mg absolute day 1–5), she achieved a partial remission, but showed progressive disease after completing six cycles CHOP-21 with new enlarged cervical lymph nodes on both sides of the neck. The patient was scheduled for myeloablative chemotherapy with autologous stem cell transplant using a DHAP protocol (cisplatin 100 mg/m^2^ day 1, cytarabine 2.000 mg/m^2^ bid day 2, and dexamethasone 40 mg absolute days 1–4) for tumor reduction and stem cell collection. Following two courses of DHAP, the patient again had progressive disease showing further increase of the cervical lymph nodes and new supraclavicular lymph nodes. A third line chemotherapy regimen with DexaBEAM (dexamethasone 8 mg absolute TID day 1–10, carmustine 60 mg/m^2^ day 2, melphalan 20 mg/m^2^ day 3, etoposide 75 mg/m^2^ day 4–7, and cytarabine 100 mg/m^2^ BID day 4–7) did not result in lymphoma remission; due to chemo-refractory disease, the myeloablative concept was abandoned (Figure 1).

**Figure 2:**
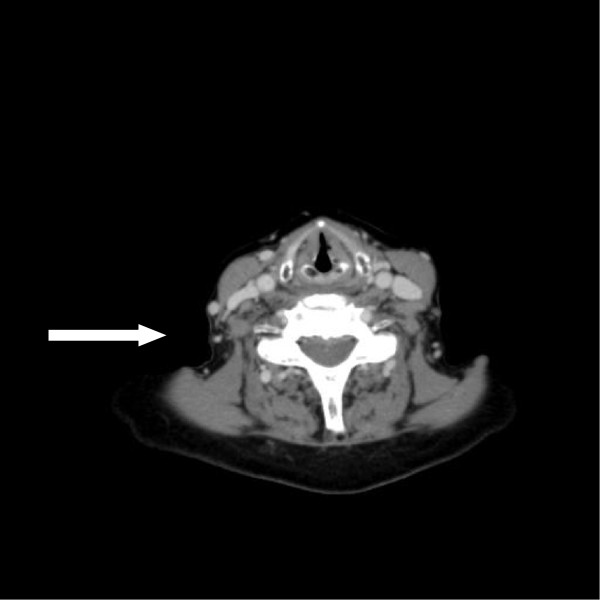
CT scan of the neck after 2 cycles of belinostat (April 2010) showing a complete response.

The patient was enrolled in January 2010 in the multicenter, open-label BELIEF-trial (Phase II study PXD101-CLN-19). Belinostat was started at a dose of 1000 mg/m^2^ for days 1–5 in a 21 day cycle. In April 2010 following two cycles of belinostat, the patient achieved a complete response (CR, Figure 2). After 1 year of belinostat therapy (~ 12 cycles), the interval between cycles was extended from 21 days to 42 days due to the long lasting remission, and to avoid potential toxicities. The patient has received a total of 28 cycles with the last cycle completed in December 2012, and maintains a CR at her last follow-up in July 2013.

In general, belinostat therapy was tolerated and administered for 28 cycles. Hematologic side effects were mild without any toxicity more than CTC grade 2. However, after cycle 2 the patient developed > 2 grade non-hematologic toxicity: CTC grade 3 relapsing bipulmonary alveolitis which was successfully treated with steroids: CTC grade 3 repeated episodes of Clostridium difficile toxin-associated diarrhoea during the first 4 months of treatment, that responded to metronidazole: CTC grade 3 antibiotic resistant atypical pneumonia while on steroid medication, that responded to treatment with voriconacole, suggesting pulmonary fungal infection. All of the non-hematologic adverse events except for the alveolitis were not considered related to belinostat treatment.

## Conclusions

Here we report a patient with chemo-refractory PTCL, status post 2 cycles of CHOP-21, 2 cycles of DHAP, and 1 cycle of DexaBEAM, who achieved a remarkably long lasting CR (39 months) after treatment with the novel HDAC inhibitor, belinostat. After attaining a CR following 2 cycles of belinostat and receiving a total of 28 cycles, the patient has remained disease-free for 8 months after treatment discontinuation. To date, this is by far the longest period a patient has been treated with belinostat, and is the first report of durable response with this agent in relapsed or refractory PTCL.

### Consent

Written informed consent was obtained from the patient for publication of this Case report and any accompanying images. A copy of the written consent is available for review by the Editor-in-Chief of this journal.

## Abbreviations

ALK: Anaplastic lymphoma kinase; BID: Twice a day [bis in die]; CHOP: Cyclophosphamide, vincristine, doxorubicin, and prednisolone; CR: Complete response; DexaBEAM: Dexamethasone, carmustine, melphalan, etoposide, and cytarabine; DHAP: Cisplatin, cytarabine, and dexamethasone; HDAC: Histone deacetylase; IPI: International prognostic index; NHL: Non-Hodgkin’s lymphomas; OS: Overall survival; PIT: Prognostic index for peripheral T-cell lymphoma; PTCL: Peripheral T/NK-cell lymphomas; PTCL-NOS: PTCL not otherwise specified; TID: Three times a day [ter in die].

## Competing interests

Peter Reimer: The author declares that he has no competing interests.

Shanta Chawla: The author is an employee and Stock holder of Spectrum Pharmaceuticals.

## Authors’ contribution

PR treated the patient and wrote the manuscript. SC participated in the design of the trial and helped to draft the manuscript. Both authors read and approved the final manuscript.
